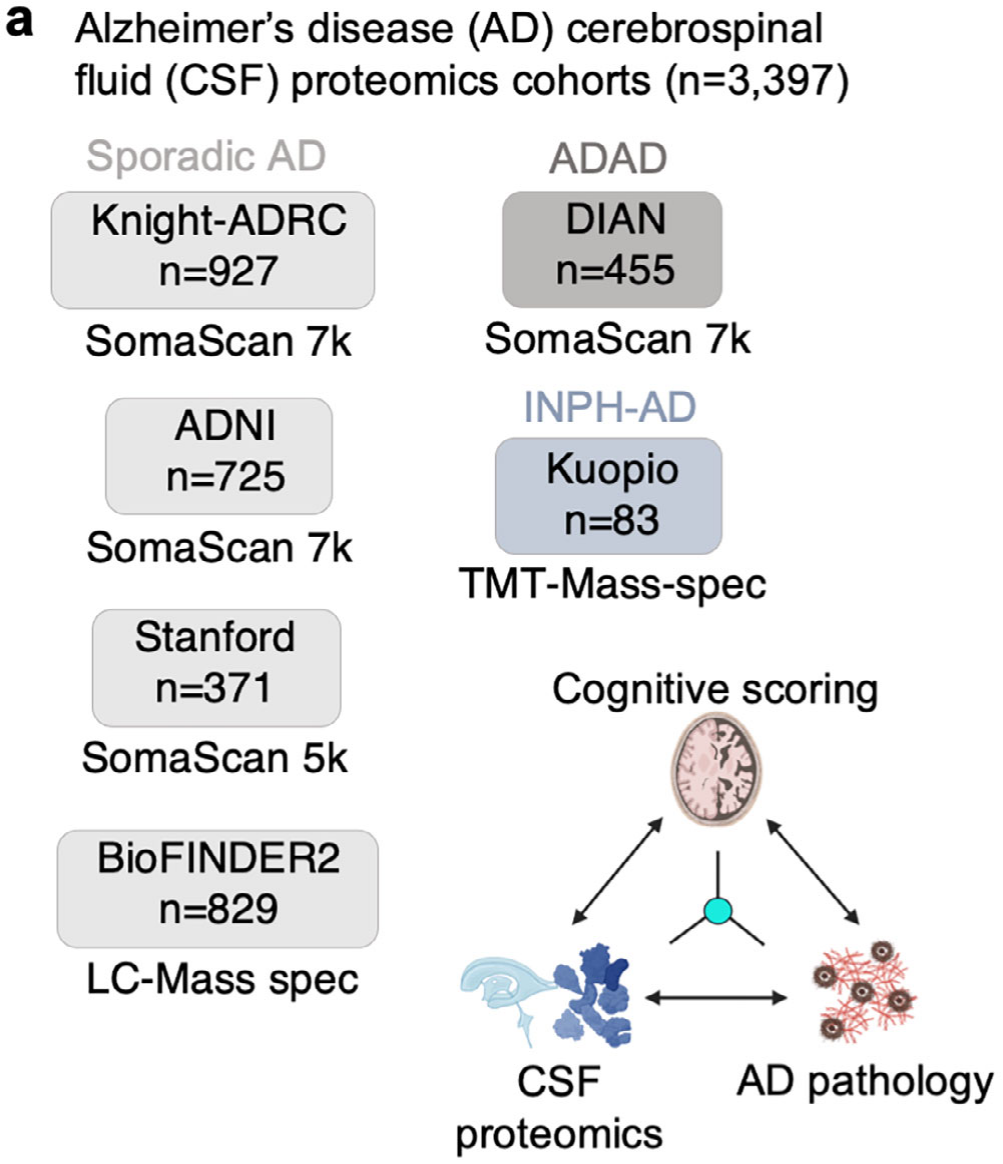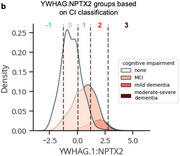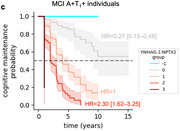# Synapse Protein Signatures in Cerebrospinal Fluid and Plasma Predict Cognitive Maintenance vs Decline in Alzheimer's Disease

**DOI:** 10.1002/alz70856_105736

**Published:** 2026-01-08

**Authors:** Tony Wyss‐Coray, Hamilton Se‐Hwee Oh

**Affiliations:** ^1^ Wu Tsai Neurosciences Institute, Stanford University, Stanford, CA, USA; ^2^ Icahn School of Medicine at Mount Sinai, New York, NY, USA

## Abstract

**Background:**

Rates of cognitive decline in Alzheimer's disease (AD) are extremely heterogeneous, with symptom onset occurring between ages 40‐100 years and conversion from mild cognitive impairment (MCI) to AD dementia occurring in 2‐20 years. Biomarkers for amyloid‐beta (Aβ) and tau proteins, the hallmark AD pathologies, have improved diagnosis and drug development but explain only 20‐40% of the variance in AD‐related cognitive impairment (CI).

**Method:**

To discover additional biomarkers of CI in AD, we perform cerebrospinal fluid (CSF) proteomics on 3,397 individuals from six major prospective AD case‐control cohorts. Synapse proteins emerge as the strongest correlates of CI, independent of Aβ and tau.

**Result:**

Using machine learning, we derive the CSF YWHAG:NPTX2 synapse protein ratio, which explains 27% of the variance in CI beyond CSF PTau181:Aβ42, 10% beyond tau PET, and 28% beyond CSF NfL, GAP43, and Ng in Aβ‐ and phosphorylated tau‐ positive (A+T_1_+) individuals. We find YWHAG:NPTX2 also increases with normal aging and at a faster rate in *APOE4* carriers and autosomal dominant‐AD mutation carriers. For prognosis, we define YWHAG:NPTX2 thresholds to stratify A+T_1_+ individuals into five groups that track with future cognitive resilience versus decline. Most notably, among A+T_1_+ MCI individuals, those in the predicted cognitively normal group have a 73% reduced risk of cognitive decline, while those in the predicted dementia group have a 2.3 times increased risk, after adjusting for CSF PTau181:Aβ42, CSF NfL, CSF Ng, CSF GAP43, age, *APOE4*, and sex. Lastly, we develop a plasma proteomic signature of CI, which we evaluate in 13,401 samples, that partly recapitulates CSF YWHAG:NPTX2.

**Conclusion:**

Overall, our findings underscore CSF YWHAG:NPTX2 and the corresponding plasma signature as robust prognostic biomarkers for AD onset and progression beyond gold‐standard biomarkers of Aβ, tau, and neurodegeneration and implicate synapse dysfunction as a core driver of AD dementia.